# Buffering brain aging: education moderates language impairment in Parkinson's disease

**DOI:** 10.3389/fncel.2025.1606451

**Published:** 2025-08-20

**Authors:** Minchul Kim, Kwangsun Yoo

**Affiliations:** ^1^Department of Radiology, Kangbuk Samsung Hospital, Sungkyunkwan University School of Medicine, Seoul, Republic of Korea; ^2^Department of Digital Health, Samsung Advanced Institute for Health Sciences and Technology (SAIHST), Sungkyunkwan University, Seoul, Republic of Korea; ^3^AI Research Center, Data Science Research Institute, Research Institute for Future Medicine, Samsung Medical Center, Seoul, Republic of Korea; ^4^Center for Neuroscience Imaging Research, Institute for Basic Science (IBS), Suwon, Republic of Korea

**Keywords:** brain age, Parkinson's disease, education, moderation analyses, cognitive reserve, language function

## Abstract

**Background:**

Cognitive reserve (CR) refers to the discrepancy between brain pathology and observed cognitive decline. While education is a key indicator of CR, its role as a potential moderator in the relationships between brain morphology and cognitive impairments in Parkinson's disease (PD) remains unclear. This study examined whether education affects the relationship between brain age and cognitive impairments in patients with PD.

**Methods:**

Data from 58 patients with PD were analyzed using a secondary dataset from the OpenNeuro database. Participants aged ≥55 years were on stable medications and underwent standardized neuropsychological assessments. Brain age predictions were generated from T1-weighted magnetic resonance imaging (MRI) using the brainageR package, and the brain age difference (BAD) was calculated after correction for regression dilution. The moderation effect of education on the relationship between BAD and cognition was assessed using Hayes' PROCESS macro. The primary outcome was cognitive performance across six domains: attention, executive function, language, learning and memory, visuospatial ability, and global cognition.

**Results:**

Among the six domains, a significant moderation effect of education was found only for language ability (β = 0.01, *p* = 0.013, *R*^2^ = 0.20). The relationship between BAD and language was steeper at lower education levels. No statistically significant moderation was found in the remaining five domains.

**Conclusion:**

Having more years of education is associated with buffering the effects of accelerated brain aging on language ability in PD.

## Introduction

Parkinson's disease (PD) is a progressive neurodegenerative disorder that affects motor control; however, non-motor symptoms, particularly cognitive impairment, are often the debilitating aspect of PD (Christopher and Strafella, 2013; Kehagia et al., 2010).

The concept of “brain age” has emerged to comprehend the aging brain and its associated neurodegenerative processes (Eickhoff et al., 2021; Franke and Gaser, 2019). Brain age models estimate an individual's brain age based on the brain MRI data, and the individual deviation of biological “brain age” from chronological age—the brain age difference (BAD)—is widely recognized as a marker of brain health (Cole et al., 2019). As PD shows evidence of robust brain atrophy (Xu et al., 2020), several studies have investigated brain age in this context (Beheshti et al., 2019; Eickhoff et al., 2021; Teipel et al., 2024). Recently, using the Parkinson's Progression Markers Initiative database, a study discovered that higher BAD in PD is primarily associated with global gray matter volume and with the basal forebrain, which is atrophied in both PD and Alzheimer's disease (Teipel et al., 2024). Interestingly, the magnitude of accelerated brain aging in patients with PD is constantly reported to be associated not only with motor but also with cognitive impairment (Chen et al., 2024; Eickhoff et al., 2021).

Meanwhile, the cognitive reserve (CR) theory seeks to explain the observed mismatch between the degree of brain pathology and clinical manifestations (Hindle et al., 2016). Indirect proxies are commonly used to measure CR, such as years of education, intelligence quotient, occupational attainment, and cognitive lifestyles (Hindle et al., 2017; Loftus et al., 2021; Stern, 2009). Previous studies discovered that years of education may protect against cognitive impairment associated with PD (Hindle et al., 2016; Loftus et al., 2021). Reportedly, higher educational attainment is associated with improved baseline cognitive performance across various domains in patients with PD (Gu and Xu, 2022). Therefore, education may contribute to a higher CR, providing individuals with a greater cognitive capacity and helping to buffer against the impact of neurodegeneration.

Based on the findings from studies explaining cognitive decline in PD in terms of BAD and CR, we hypothesized that higher years of education might modulate the cognitive decline associated with neurodegeneration in PD. Specifically, the study explored whether years of education moderate the relationship between BAD and multiple domains of cognitive decline in PD.

## Materials and methods

### Patient selection and demographics

**Figure 1:**
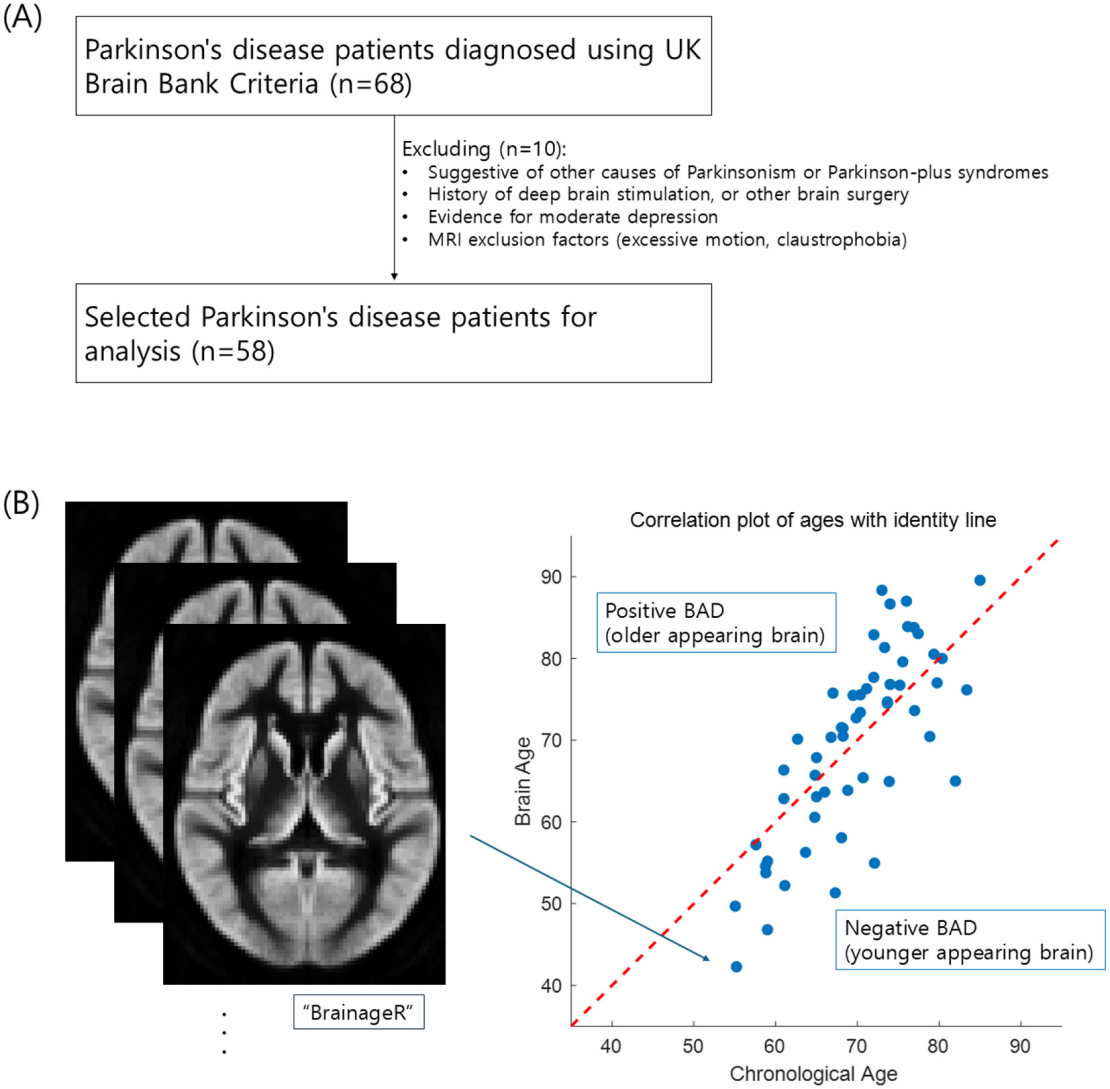
Flow diagram of data acquisition **(A)** and the scatterplot of brain age predictions **(B)** for patients with Parkinson's disease. The red diagonal line in **(B)** represents a perfect prediction accuracy. Dots above the line represent a predicted brain age older than the chronological age, while those below represents a predicted brain age younger than the chronological age (Pearson's *r* = 0.769, *p* < 0.001).

This study used secondary data from an open-access dataset available on OpenNeuro at https://openneuro.org/datasets/ds004392/versions/1.0.0 (Wylie et al., 2023). Sixty-eight patients with PD were recruited through the University of Colorado Hospital's Movement Disorder, Memory Disorder, and Neuropsychology Clinics. Diagnosis of PD was defined using UK Brain Bank Criteria (Hughes et al., 1992). All participants provided informed consent to participate in the study, which was approved by the Colorado Multiple Institution Review Board and conducted in accordance with the Declaration of Helsinki (Wylie et al., 2023). According to the original study from which the data were pooled, the exclusion criteria included features suggestive of other causes of parkinsonism or Parkinson-plus syndromes; features suggestive of other causes of dementia, including moderate to severe cerebrovascular disease by history or imaging; history of major head trauma; history of deep brain stimulation, ablation surgery, or other brain surgery; evidence of moderate depression based on the Hospital Anxiety Depression Scale; and MRI exclusion factors (Figure 1A) (Wylie et al., 2023). Finally, 58 participants were included after applying the selection criteria.

All patients underwent a comprehensive battery of neuropsychological tests, including the Montreal Cognitive Assessment, Mattis Dementia Rating Scale 2, Trail Making Test Parts A and B, Brief Test of Attention, Boston Naming Test (BNT), Verbal Phonemic Fluency (FAS), California Verbal Learning Test Second Edition, Judgment of Line Orientation (JLO), and the Symbol Digit Modality Test. These tests were chosen based on previous study that validated the PD-MCI diagnostic criteria (Goldman et al., 2013). From the full battery of tests, five cognitive domains (attention, executive function, language, learning and memory, and global cognition) were defined using principal component analysis (PCA), with raw scores from two tests put into each conceptual component, e.g., BNT and FAS for the “language” domain. Finally, the visuospatial domain, derived from a sample-based z-score of JLO scores, completed the set of six cognitive domains analyzed in this study (Wylie et al., 2023).

### MRI acquisition, quality control, and brain age estimation

MRI scans were performed using a 3.0 T Signa scanner (GE Healthcare, Milwaukee, WI) with an eight-channel head coil and a 3D inversion recovery spoiled gradient-recalled echo imaging sequence with a dynamic range. The structural scans were acquired with the following parameters: TR = 2,200 ms, TE = 2 ms, matrix = 256 × 256, voxel size = 1 × 1 mm^2^, slice thickness = 1 mm, and flip angle = 8°. All first-level datasets were visually inspected to ensure data quality.

Brain age was estimated using brainageR (v2.1), open-access software for generating brain-predicted age from raw T1-weighted MRI scans (https://github.com/james-cole/brainageR) (Cole et al., 2019). BrainageR primarily involves the preprocessing and prediction stages. In preprocessing, the images are segmented and normalized via SPM12 (https://www.fil.ion.ucl.ac.uk/spm/software/spm12/). For quality control, the FSL slicesdir function was used to generate two-dimensional slices of the segmentation and normalization outputs. Next, normalized images were loaded into R and converted to vectors (R Core Team, 2013). Using a 0.3 threshold from the mean image template based on the brainageR model training dataset, gray matter, white matter, and cerebrospinal fluid vectors were masked and combined. In prediction, the brainageR model was applied to vectorized and masked study images to estimate a brain age score for each. BrainageR had been previously trained to predict age from normalized brain volumetric maps in 3,377 healthy individuals from 7 publicly available datasets using a Gaussian process regression (see Appendix for a list of training datasets) (Biondo et al., 2022). Using PCA, the top principal components capturing 80% of the variance in brain volumes were retained. The resulting rotation matrix for the 435 PCs was then applied to the new imaging data for predicting age (Biondo et al., 2022).

After calculating the predicted brain age for each subject, BAD was calculated. BAD was initially measured by subtracting the true brain age from the predicted brain age; a higher BAD implies an older brain morphology compared to the chronological age. Owing to regression dilution, regression models may bias the predicted brain age toward the mean, underestimating and overestimating the ages of older and younger participants, respectively. Herein, to rectify this bias, BAD has been defined as the difference between individual and expected BADs (measurement fitted over the entire sample set using the regression model and leave-one-out cross-validation) (Kang et al., 2023).

### Statistical analysis

The collected data were analyzed using R version 4.4.3 (R Core Team, 2013). Descriptive statistics were used to characterize the sample demographics and the correlation between BAD and the six cognitive domains studied. Finally, the study examined the moderating or interaction effect of years of education (moderator, W) on the relationship between BAD (continuous, X) and cognitive measures (continuous, Y), with the Hayes PROCESS Macro Ver. 4.1 (Hayes, 2017). False discovery rate (FDR) correction, as well as the Hommel method, were applied to consider multiple comparisons of these six moderation models (Benjamini and Hochberg, 1995; Hommel, 1988). The sample size was additionally determined through power analysis using G^*^Power software (Faul et al., 2007). With a medium effect size (*f*^2^ = 0.15), a power level of 0.80, and an alpha level of 0.05, the calculation indicated that 55 participants were required to achieve sufficient statistical power. Our study included 58 participants, which closely approximate the calculated requirements.

## Results

Table 1 and Figure 1 present the characteristics of the study participants and the brain age estimation process. As shown in the scatter plot in Figure 1B, brain age tends to increase with chronological age, with a Pearson's correlation of *r* = 0.769 (*p* < 0.001). The mean absolute error of brain age prediction is 6.05 ± 4.48. There was variability in antiparkinsonian medication doses, which may affect cognitive performance and limit the generalizability of the results.

**Table 1:** Clinical characteristics of the study population.

**Variable**	**Mean (SD)**	**Range (min:max)**
Sex (male and female)	*n* = 39, *n* = 19	
Age	70.42 ± 7.94	55:89
Education	16.21 ± 2.75	
H and Y stage	2.71 ± 1.05	0:5
UPDRS part III	22.43 ± 8.8	0:43
LEDD (mg)	530.28 ± 431.84	0:2,100
Predicted brain age	70.62 ± 11.45	42.27:89.56
Brain age mean absolute error	6.05 ± 4.48	0.079:16.79

Values indicate mean ± SD unless otherwise indicated. LEDD, levodopa equivalent daily dose; H and Y, Hoehn and Yahr stage; UDPRS-III, Unified Parkinson's Disease Rating Scale.

Among the six cognitive domains, only the moderation model with language ability as the dependent variable showed model significance with an FDR- and the Hommel-adjusted *p*-value of 0.0492 (raw *p* = 0.0093 for language), which means that BAD, years of education, and their interaction explain a relevant portion of the variance in language ability. A sensitivity analysis using Bayesian estimation revealed a Bayes factor of 4.60, indicating moderate evidence in favor of the alternative hypothesis over the null hypothesis (Van Doorn et al., 2021). The model had an *R*^2^ of 0.2001, indicating that this model accounted for approximately 20.01% of the language score variation. The remaining five domains—global cognition (*R*^2^ = 0.12, Hommel-adjusted *p* = 0.17), attention (*R*^2^ = 0.04, adjusted *p* = 0.51), learning and memory (*R*^2^ = 0.11, adjusted *p* = 0.23), executive (*R*^2^ = 0.15, adjusted *p* = 0.15), and visuospatial (*R*^2^ = 0.18, adjusted *p* = 0.08)—did not yield significant moderation effects after multiple comparison correction.

**Figure 2:**
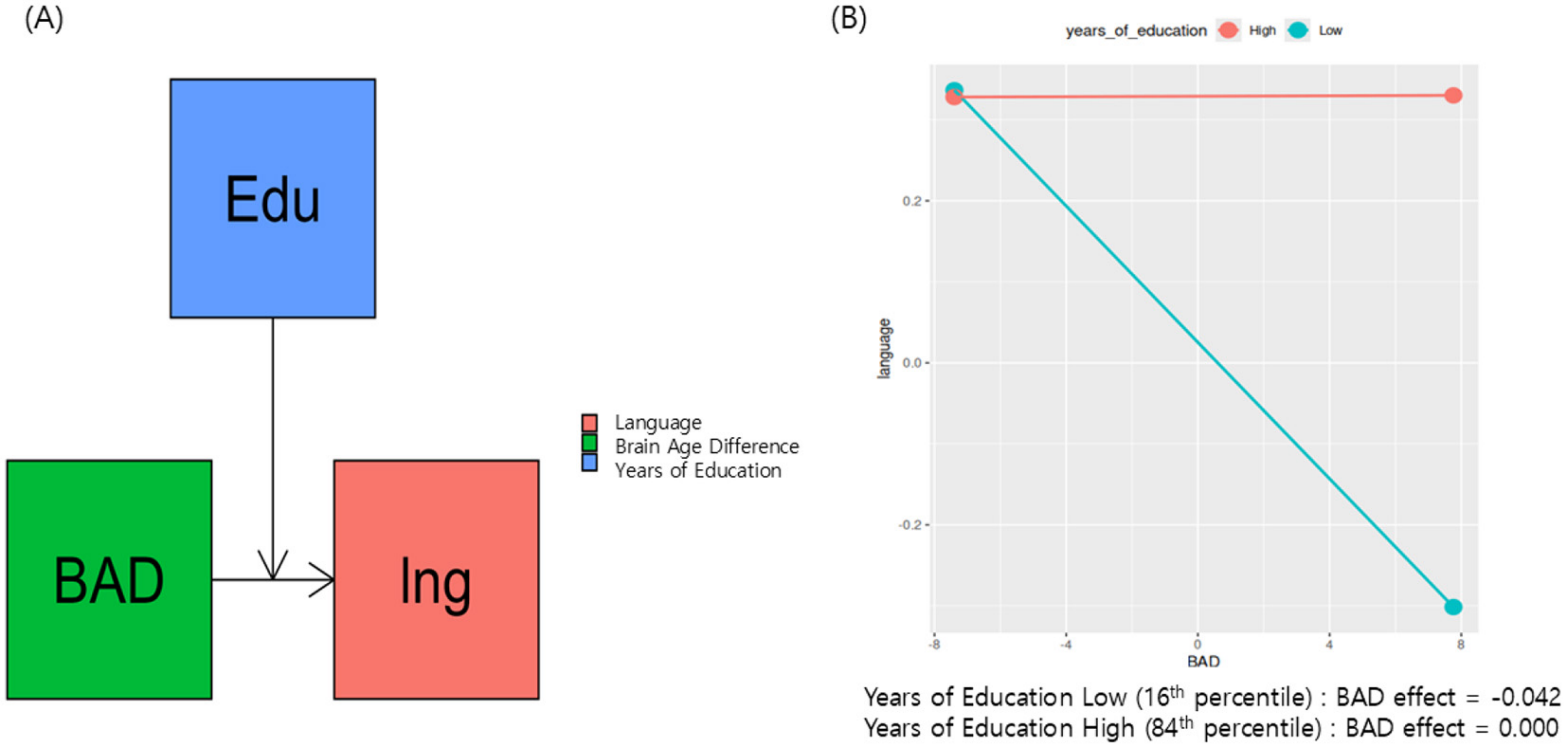
Proposed research model presents education as a moderator of brain aging and cognitive decline in Parkinson's disease **(A)**. **(B)** Shows that, at the 16th percentile (having 13.96 years of education), the effect of BAD on language ability was 0.042 and statistically significant (*p* = 0.014).

Notably, the moderation model with language as the dependent variable had significant model coefficients (Table 2, Figure 2A). There was a significant interaction between years of education and BAD [β (95% CI) = 0.0106 (0.0023–0.0188)]. Furthermore, we examined the effect of BAD on language changes at different education levels—specifically at the 16th, 50th, and 84th percentile ranks—within the sample of 58 subjects. At the 16th percentile (having 13.96 years of education), the effect of BAD on language ability was 0.0425 and statistically significant (*p* = 0.0141, Figure 2B); this finding indicates that, for participants with low years of education, a high BAD was associated with a significant decrease in language scores.

**Table 2:** Characteristics of the model using education as a moderator of brain aging and language ability.

**Predictors**	**Unstandardized coefficients**	**SE**	***t***	***p***	**95% CI**	
					**Lower**	**Upper**
**Model (***R*^2^ = **0.2001**, *F*_(3.51)_ = **4.2523**, ***p*** = **0.0093)**						
BAD	−0.1899	0.0701	−2.7092	0.0092	−2.1113	−0.0492
Education	0.0760	0.0328	2.3193	0.0244	0.0102	0.1417
Interaction	0.0106	0.0041	2.5709	0.0131	0.0023	0.0188

## Discussion

This study explored whether CR moderates the interaction between neurodegeneration and the multiple domains of cognitive decline in PD, using years of education as a proxy for CR and BAD as a marker of neurodegeneration. After multiple comparison corrections, only the moderation model with language as the dependent variable was found to be significant. These results support the hypothesis that years of education moderate the relationship between BAD and language ability decline (Table 2 and Figure 2A). For individuals with fewer years of education, a higher BAD (Figure 1B, older appearing brain) was associated with lower language scores (Figure 2B, effect = −0.042). With increasing years of education, the negative association between BAD and language scores weakens and becomes non-significant at higher education years (Figure 2B, effect = 0.000). These findings provide evidence for a nuanced relationship, in which the impact of brain aging on language is not uniform but influenced by the educational background, and emphasize the benefit of achieving basic years of education. Similarly, Glatt et al. reported that having less than a high school diploma of education considerably predicts dementia in PD, although the relationship between years of education and the dementia rating scale score was small (*r* = 0.12) (Glatt et al., 1996). Altogether, the education's buffering effect against cognitive decline is more effective in achieving basic years of education.

Furthermore, we identified a single significant moderation model explaining language ability after multiple comparison correction. The cognitive tasks measuring language ability (i.e., BNT, FAS) focus on verbal fluency. Previous studies investigating the effect of education on verbal fluency reported that education has a positive effect on this language ability. Loftus et al. investigated the relationship between CR and cognitive decline in a large PD sample (*n* = 334) and reported that years of education significantly predicted verbal fluency measured by the FAS (Loftus et al., 2021). This task requires participants to generate as many words as possible within a set time, according to specific rules (Sauzéon et al., 2011). Task performance is, therefore, dependent on one's processing speed, executive functions, and vocabulary level. Accordingly, having higher years of education and vocabulary offsets the effects of cognitive decline associated with aging on letter fluency tasks (Sauzéon et al., 2011). In a study of 1,392 patients with progressive cognitive decline, Zamarian et al. reported that, although higher education is not helpful for episodic memory and executive functions at low cognitive levels, it is beneficial for retrieving words or other semantic knowledge (Zamarian et al., 2021). Therefore, the protective effect of education might be nuanced and could vary depending on the specific cognitive functions under examination. These inconsistencies highlight the interplay complexity between education and cognitive decline in the context of PD and suggest that BAD could be the key to explaining such inconsistencies and complexity.

Previous literature identified multiple gray matter regions, including the bilateral hippocampi, the right inferior temporal gyrus, and the right lateral orbitofrontal gyrus, as images feature attributable to advanced brain aging in PD (Eickhoff et al., 2021). These striato-prefrontal brain areas, driving accelerated brain aging, were demonstrated to have functional effects on cognition in the patient group (Chen et al., 2024). On the other hand, education may induce structural brain changes via neuroplasticity, making it a promising modifiable factor in PD intervention strategies (Arenaza-Urquijo et al., 2017a,b). Having a cognitive lifestyle, including longer education, is linked to neurotrophic changes in the prefrontal lobe, implying a compensatory process (Bennett et al., 2014). Longer education not only enhances the cognitive ability *per se*, but also compensates for brain structure that may contribute to preserving language ability, as evidenced by better verbal language ability showing greater structural covariance between left and right frontal regions (Qi et al., 2019). In addition, memory in the brain can be broadly categorized into two systems: explicit memory, which involves the conscious recall of people, places, and objects, and implicit memory, which underlies automatic skills such as driving or using correct grammar (Kandel, 2018; Squire et al., 1993). Unlike explicit memory, which depends heavily on higher cognitive regions—primarily the neocortex and hippocampus, both of which are commonly implicated in brain age models—implicit memory relies more on subcortical and sensorimotor-related structures, such as the amygdala and cerebellum (Teipel et al., 2024). Therefore, cognitive tasks that engage explicit memory and language may be more vulnerable to brain aging effects, explaining why language ability showed a significant moderation effect in this study.

There are some limitations to this study. First, our dataset of 58 PD subjects lacks a healthy comparison control group, longitudinal follow-up, and diversity in the sample (e.g., variability in education and ethnicity), as well as possible confounding effects from antiparkinsonian medications, which may inflate the model estimates and limit the generalizability of the results. Second, potential confounding factors, such as lifestyle and coexisting medical conditions, which can influence brain age and cognitive function, can make it challenging to isolate the specific effects of education on brain age and cognitive decline in PD research. Third, the sample shows a gender imbalance with more male participants, which reflects the known higher prevalence of Parkinson's disease in men (Wooten et al., 2004). However, we acknowledge that this may still limit the generalizability of our findings regarding sex-specific effects. Finally, proxies of CR that may explain cognition other than years of education were not evaluated. For example, Koerts et al. examined the relationship between CR and cognition using years of education and premorbid IQ as CR proxies and reported that premorbid IQ predicted executive function in PD, but not years of education (Koerts et al., 2011).

In conclusion, this study highlights the role of education in buffering the cognitive decline associated with accelerated brain aging in PD, particularly when referring to language abilities. Therefore, interventions promoting basic level educational achievements may help mitigate cognitive impairment in patients with PD.

## Data Availability

The original contributions presented in the study are included in the article/supplementary material, further inquiries can be directed to the corresponding author.

## Appendix

The brainageR model for v2.1 was trained on n = 3,377 healthy individuals (mean age = 40.6 years, SD = 21.4, age range 18–92 years) from seven publicly available datasets (see list below).

Training datasets:

- Australian Imaging, Biomarker and Lifestyle Flagship Study of Ageing (AIBL)
- Dallas Lifespan Brain Study (DLBS)
- Brain Genome Superstruct Project (GSP)
- IXI
- Nathan Kline Institute Rocklands Sample Enhanced
- Open Acces Series of Imaging Studies-1 (OASIS-1)
- Southwest University Adult Lifespan Dataset (SALD)

## References

[B1] Arenaza-Urquijo E. M. Bejanin A. Gonneaud J. Wirth M. La Joie R. Mutlu J.. (2017a). Association between educational attainment and amyloid deposition across the spectrum from normal cognition to dementia: neuroimaging evidence for protection and compensation. Neurobiol. Aging 59, 72–79. 10.1016/j.neurobiolaging.2017.06.016 28764930

[B2] Arenaza-Urquijo E. M. de Flores R. Gonneaud J. Wirth M. Ourry V. Callewaert W.. (2017b). Distinct effects of late adulthood cognitive and physical activities on gray matter volume. Brain Imaging Behav. 11, 346–356. 10.1007/s11682-016-9617-3 27757821

[B3] Beheshti I. Mishra S. Sone D. Khanna P. Matsuda H. (2019). T1-weighted MRI-driven brain age estimation in Alzheimer's disease and Parkinson's disease. Aging Dis. 11:618. 10.14336/AD.2019.0617 32489706 PMC7220281

[B4] Benjamini Y. Hochberg Y. (1995). Controlling the false discovery rate: a practical and powerful approach to multiple testing. J. R. Stat. Soc. Ser. B 57, 289–300. 10.1111/j.2517-6161.1995.tb02031.x

[B5] Bennett D. A. Arnold S. E. Valenzuela M. J. Brayne C. Schneider J. A. (2014). Cognitive and social lifestyle: links with neuropathology and cognition in late life. Acta Neuropathol. 127, 137–150. 10.1007/s00401-013-1226-2 24356982 PMC4054865

[B6] Biondo F. Jewell A. Pritchard M. Aarsland D. Steves C. J. Mueller C.. (2022). Brain-age is associated with progression to dementia in memory clinic patients. Neuroimage Clin. 36 :103175. 10.1016/j.nicl.2022.103175 36087560 PMC9467894

[B7] Chen C.-L. Cheng S.-Y. Montaser-Kouhsari L. Wu W.-C. Hsu Y.-C. Tai C.-H.. (2024). Advanced brain aging in Parkinson's disease with cognitive impairment. NPJ Parkinsons Dis. 10:62. 10.1038/s41531-024-00673-7 38493188 PMC10944471

[B8] Christopher L. Strafella A. P. (2013). Neuroimaging of brain changes associated with cognitive impairment in Parkinson's disease. J. Neuropsychol. 7, 225–240. 10.1111/jnp.12015 23551844 PMC4452222

[B9] Cole J. H. Marioni R. E. Harris S. E. Deary I. J. (2019). Brain age and other bodily ‘ages': implications for neuropsychiatry. Mol. Psychiatry 24, 266–281. 10.1038/s41380-018-0098-1 29892055 PMC6344374

[B10] Eickhoff C. R. Hoffstaedter F. Caspers J. Reetz K. Mathys C. Dogan I.. (2021). Advanced brain ageing in Parkinson's disease is related to disease duration and individual impairment. Brain Commun. 3:fcab191. 10.1093/braincomms/fcab191 34541531 PMC8445399

[B11] Faul F. Erdfelder E. Lang A.-G. Buchner A. (2007). G^*^ Power 3: a flexible statistical power analysis program for the social, behavioral, and biomedical sciences. Behav. Res. Methods 39, 175–191. 10.3758/BF03193146 17695343

[B12] Franke K. Gaser C. (2019). Ten years of BrainAGE as a neuroimaging biomarker of brain aging: what insights have we gained? Front. Neurol. 10:789. 10.3389/fneur.2019.00789 31474922 PMC6702897

[B13] Glatt S. L. Hubble J. P. Lyons K. Paolo A. (1996). Risk factors for dementia in Parkinson's disease: effect of education. Neuroepidemiology 15, 20–25. 10.1159/000109885 8719045

[B14] Goldman J. G. Holden S. Bernard B. Ouyang B. Goetz C. G. Stebbins G. T. (2013). Defining optimal cutoff scores for cognitive impairment using Movement Disorder Society Task Force criteria for mild cognitive impairment in Parkinson's disease. Mov. Disord. 28, 1972–1979. 10.1002/mds.25655 24123267 PMC4164432

[B15] Gu L. Xu H. (2022). Effect of cognitive reserve on cognitive function in Parkinson's disease. Neurol. Sci. 43, 4185–4192. 10.1007/s10072-022-05985-1 35230598

[B16] Hayes A. F. (2017). Introduction to Mediation, Moderation, and Conditional Process Analysis: A Regression-Based Approach. Guilford publications.

[B17] Hindle J. V. Hurt C. S. Burn D. J. Brown R. G. Samuel M. Wilson K. C.. (2016). The effects of cognitive reserve and lifestyle on cognition and dementia in Parkinson's disease—a longitudinal cohort study. Int. J. Geriatr. Psychiatry 31, 13–23. 10.1002/gps.4284 25781584

[B18] Hindle J. V. Martin-Forbes P. A. Martyr A. Bastable A. J. Pye K. L. Mueller Gathercole V. C.. (2017). The effects of lifelong cognitive lifestyle on executive function in older people with Parkinson's disease. Int. J. Geriatr. Psychiatry 32, e157–e165. 10.1002/gps.4677 28170111

[B19] Hommel G. (1988). A stagewise rejective multiple test procedure based on a modified Bonferroni test. Biometrika 75, 383–386. 10.1093/biomet/75.2.383

[B20] Hughes A. J. Daniel S. E. Kilford L. Lees A. J. (1992). Accuracy of clinical diagnosis of idiopathic Parkinson's disease: a clinico-pathological study of 100 cases. J. Neurol. Neurosurg. Psychiatry 55, 181–184. 10.1136/jnnp.55.3.181 1564476 PMC1014720

[B21] Kandel E. R. (2018). The Disordered Mind: What Unusual Brains Tell Us About Ourselves. Hachette UK.

[B22] Kang S. H. Liu M. Park G. Kim S. Y. Lee H. Matloff W.. (2023). Different effects of cardiometabolic syndrome on brain age in relation to gender and ethnicity. Alzheimers Res. Ther. 15:68. 10.1186/s13195-023-01215-8 36998058 PMC10061789

[B23] Kehagia A. A. Barker R. A. Robbins T. W. (2010). Neuropsychological and clinical heterogeneity of cognitive impairment and dementia in patients with Parkinson's disease. Lancet Neurol. 9, 1200–1213. 10.1016/S1474-4422(10)70212-X 20880750

[B24] Koerts J. Van Beilen M. Tucha O. Leenders K. L. Brouwer W. H. (2011). Executive functioning in daily life in Parkinson's disease: initiative, planning and multi-task performance. PLoS ONE 6:e29254. 10.1371/journal.pone.0029254 22206004 PMC3243690

[B25] Loftus A. M. Gasson N. Lopez N. Sellner M. Reid C. Cocks N.. (2021). Cognitive reserve, executive function, and memory in Parkinson's disease. Brain Sci. 11:992. 10.3390/brainsci11080992 34439609 PMC8391924

[B26] Qi T. Schaadt G. Cafiero R. Brauer J. Skeide M. A. Friederici A. D. (2019). The emergence of long-range language network structural covariance and language abilities. Neuroimage 191, 36–48. 10.1016/j.neuroimage.2019.02.014 30738206

[B27] R Core Team (2013). R: A Language and Environment for Statistical Computing. R Core Team.

[B28] Sauzéon H. Raboutet C. Rodrigues J. Langevin S. Schelstraete M.-A. Feyereisen P.. (2011). Verbal knowledge as a compensation determinant of adult age differences in verbal fluency tasks over time. J. Adult Dev. 18, 144–154. 10.1007/s10804-010-9107-6

[B29] Squire L. R. Knowlton B. Musen G. (1993). The structure and organization of memory. Annu. Rev. Psychol. 44, 453–495. 10.1146/annurev.ps.44.020193.002321 8434894

[B30] Stern Y. (2009). Cognitive reserve. Neuropsychologia 47, 2015–2028. 10.1016/j.neuropsychologia.2009.03.004 19467352 PMC2739591

[B31] Teipel S. J. Hoffmann H. Storch A. Hermann A. Dyrba M. Schumacher J. (2024). Brain age in genetic and idiopathic Parkinson's disease. Brain Commun. 6:fcae382. 10.1093/braincomms/fcae382 39713239 PMC11660940

[B32] Van Doorn J. Van Den Bergh D. Böhm U. Dablander F. Derks K. Draws T.. (2021). The JASP guidelines for conducting and reporting a Bayesian analysis. Psychon. Bull. Rev. 28, 813–826. 10.3758/s13423-020-01798-5 33037582 PMC8219590

[B33] Wooten G. Currie L. Bovbjerg V. Lee J. Patrie J. (2004). Are men at greater risk for Parkinson's disease than women? J. Neurol. Neurosurg. Psychiatry 75, 637–639. 10.1136/jnnp.2003.020982 15026515 PMC1739032

[B34] Wylie K. Kluger B. Medina L. Holden S. Kronberg E. Tregellas J.. (2023). Parkinson's Disease, Functional Connectivity, and Cognition. OpenNeuro. 10.1111/ejn.15899 PMC9970048 36516060

[B35] Xu X. Han Q. Lin J. Wang L. Wu F. Shang H. (2020). Grey matter abnormalities in Parkinson's disease: a voxel-wise meta-analysis. Eur. J. Neurol. 27, 653–659. 10.1111/ene.14132 31770481

[B36] Zamarian L. Karner E. Bodner T. Djamshidian A. Delazer M. (2021). Differential impact of education on cognitive performance in neurological patients with progressive cognitive decline. J. Alzheimers Dis. 80, 1491–1501. 10.3233/JAD-201608 33720899

